# Two Pathways to Self-Harm in Adolescence

**DOI:** 10.1016/j.jaac.2021.03.010

**Published:** 2021-12

**Authors:** Stepheni Uh, Edwin S. Dalmaijer, Roma Siugzdaite, Tamsin J. Ford, Duncan E. Astle

**Affiliations:** a Ms. Uh and Drs. Dalmaijer, Siugzdaite, and Astle are with MRC Cognition and Brain Sciences Unit, University of Cambridge, United Kingdom; b Dr. Ford is with the University of Cambridge, United Kingdom

**Keywords:** self-harm, adolescence, socioemotional profiles, longitudinal analysis

## Abstract

**Objective:**

The behavioral and emotional profiles underlying adolescent self-harm, and its developmental risk factors, are relatively unknown. We aimed to identify subgroups of young people who self-harm (YPSH) and longitudinal risk factors leading to self-harm.

**Method:**

Participants were from the Millennium Cohort Study (N = 10,827). A clustering algorithm was used to identify subgroups who self-harmed with different behavioral and emotional profiles at age 14 years. We then traced the profiles back in time (ages 5−14 years) and used feature selection analyses to identify concurrent correlates and longitudinal risk factors of self-harming behavior.

**Results:**

There were 2 distinct subgroups at age 14 years: a smaller group (n = 379) who reported a long history of psychopathology, and a second, much larger group (n = 905) without. Notably, both groups could be predicted almost a decade before the reported self-harm. They were similarly characterized by sleep problems and low self-esteem, but there was developmental differentiation. From an early age, the first group had poorer emotion regulation, were bullied, and their caregivers faced emotional challenges. The second group showed less consistency in early childhood, but later reported more willingness to take risks and less security with peers/family.

**Conclusion:**

Our results uncover 2 distinct pathways to self-harm: a “psychopathology” pathway, associated with early and persistent emotional difficulties and bullying; and an “adolescent risky behavior” pathway, whereby risk taking and external challenges emerge later into adolescence and are associated with self-harm. At least one of these pathways has a long developmental history, providing an extended window for interventions as well as potential improvements in the identification of children at risk, biopsychosocial causes, and treatment or prevention of self-harm.

Self-harm is commonly associated with poor mental health in both clinical and nonclinical populations, with prevalence estimates ranging from approximately 13.2% to 19.7% among adolescents in England.^1^, ^2^, ^3^ The definition of self-harm varies, because of complexity in its presentation and description (eg, nonsuicidal self-harm/self-injury,^4^ deliberate self-harm^5^). For the present purposes, it is defined as the purposeful act of hurting oneself with or without suicidal intent. Self-harm is a significant risk factor for subsequent suicide attempts and, consequently, is a strong risk factor for death by suicide among adolescents.^6^ Here we define “risk factors” as factors that precede and can distinguish high- and low-risk groups for self-harm.^7^ However, many do not intend suicide and face other harmful outcomes including repetition of self-harm, various mental health issues, and risky behaviors such as substance abuse.^1^^,^^6^ Although self-harm is recognized as a global health concern, it remains a highly prevalent issue in adolescents worldwide.^2^

Despite its prevalence and lifelong consequences, there has been little progress in the accurate prediction of self-harm.^8^ This may be due to the paucity of longitudinal studies investigating early risk factors in a nonretrospective manner, especially in nonclinical samples.^9^^,^^10^ Although there is still much to uncover regarding the developmental trajectories underlying this poor mental health outcome in young people, enhanced research efforts have identified a wide range of potential self-harm risk factors. One prominent review identifies several domains of risk associated with self-harm, including sociodemographic and educational factors (eg, low socioeconomic status, female sex, restricted education), negative life events and family adversity (eg, bullying, abuse), and psychiatric and psychological challenges (eg, mental disorder, impulsivity, low self-esteem).^10^ A study of adolescents in the United Kingdom, furthermore, found that repeated self-harm was strongly linked to personality disturbances, depression, substance use, troubled relationships with peers/family, poor school performance, and chronic psychosocial as well as behavioral difficulties.^11^ In addition, both the theoretical and empirical literatures underscore early childhood adverse experiences, including sexual and/or physical abuse, neglect, and unstable emotional attachments to caregivers, as leading risk factors for self-harm.^9^ In short, the recent surge of studies has identified a multitude of internal and external risk factors for self-harm. However, we are still unable to accurately predict this outcome, which may in part be due to several empirical challenges: a large proportion of self-harm research being cross-sectional,^10^ the majority of studies being conducted in psychiatric or clinical samples,^12^ many studies using retrospective self-report data (which may cause some bias or inaccurate accounts of early childhood experiences),^9^ and studies often using statistical approaches tailored toward finding one set of risk factors, making it difficult to capture the multidimensional nature of self-harm risk.^13^^,^^14^

An added complication is the heterogeneous nature of self-harm, including potential subtypes of self-harming behavior and, importantly, different subgroups of young people who self-harm (YPSH). A large adolescent study in Germany identified 2 types of self-harming behavior in terms of frequency: (1) occasional self-harm and (2) repetitive self-harm.^14^ Although both subtypes were associated with anxiety and depressive symptoms, in addition to delinquent behavior, they found that occasional—but not repetitive—self-harm was related to external factors such as poor education and family challenges, including health problems of caregivers. In contrast, repetitive self-harm was more strongly associated with internal factors such as prior suicidal ideation and body-image issues.^14^ However, the researchers note that the cross-sectional design of this study makes it difficult to know whether these factors are risk factors or consequences of self-harm. This is made yet more complex by the possibility of different psychological subgroups or profiles of YPSH.^13^^,^^15^ Stanford *et al.,*^13^ for instance, identified 5 distinct psychological profiles of YPSH: nonpsychopathological, anxious, impulsive, pathological, and pathological−impulsive. Their results highlight the value of investigating profiles, as these subgroups may have different risk factors and pathways leading to self-harm. In addition, although the pathological profiles are in line with risk factors identified from prior research, the subgroup without obvious psychological symptoms—who also did not report problems with social support or bullying—reflects the major challenge of identifying and providing care for a large proportion of YPSH in the general community.^13^ It is likely that the risk factors that have been identified from prior research and self-harm models (eg, mental health problems, adversity) do not apply to this subgroup. Thus, using more complex models to investigate a variety of potential risk factors and risk factors associated with these YPSH subgroups and their developmental trajectories could be essential to inform more effective and targeted treatments.^13^^,^^16^

Despite the increase in self-harm research over recent decades, we have made minimal progress in addressing a key set of questions that are relevant to researchers, policy makers, and practitioners.^7^ What are the risk factors for self-harm? Will all YPSH present with a similar emotional and behavioral profile? Finally, how early in childhood do these risks emerge? The purpose of this study is to address these questions.

Previous work exploring self-harm has largely focused on those who present to hospital or other clinical facilities,^1^^,^^3^ but this is unlikely to capture all self-harming behavior. McManus *et al.,*^1^ for instance, found that self-reported lifetime nonsuicidal self-harm increased from 2.4% in 2000 to 6.4% in 2014 in England, but most individuals did not present to medical or psychological services. This highlights that those who seek help after harming themselves are likely to vary in a number of ways from those who do not seek help; thus, risk factors identified in clinically recruited samples may not be generalizable to YPSH in the population. It is not surprising, therefore, that clinical services currently available for self-harm are mostly responsive. Furthermore, although many YPSH do not seek help, those who do often face lengthy waits for a generic intervention or treatments that lack a strong evidence base.^17^ Another limitation of relying solely on clinical recruitment is that we are unable to investigate the early developmental trajectories of adolescents who ultimately self-harm in comparison to those who do not.^18^ Further exploring the existence of subgroups of YPSH in community-based samples and identifying different developmental trajectories may assist in tailoring prevention and intervention measures to make them more effective.^13^^,^^17^

In the current study, we identified adolescents who reported self-harm at age 14 years, from a nationally representative UK birth cohort of approximately 19,000 individuals. We then used a machine learning analysis to identify whether there are distinct clusters of YPSH, with different emotional and behavioral presentations. We subsequently identified the concurrent correlates using the extensive dataset available on these individuals. Finally, we used the preceding waves of data collection from when the children were 5, 7, and 11 years of age to identify risk factors from early and middle childhood.

## Method

### Participants

The Millennium Cohort Study^19^ (MCS) is a large-scale, ongoing, longitudinal developmental study of young people throughout the United Kingdom. An extensive amount of behavioral, socio-emotional, and physical data on the participants have been collected since they were 9 months of age. From the original dataset of 11,884 individuals at age 14 years, we included 10,827 (50% female) participants who had complete responses to the measures used in our subgroup analysis—namely, the Strength and Difficulties Questionnaire (SDQ)^20^ and the Mood and Feelings Questionnaire (MFQ).^21^ Within the participants who reported self-harm (n = 1,580, 73% female), our main analysis would subsequently focus on a large subset (n = 1,284, 74% female) who fit into 2 distinct behavioral clusters. We also included a random subsample of participants (n = 900, 70% female) who did not self-harm, as a comparison group for subsequent analyses. For our analyses to identify risk factors, a “train” sample (70% of total) was taken from each group and used to fit our model, whereas each held-out “test” sample (30%) was used to validate the risk factors. All samples were randomly selected using R version 3.6.2 (R Project for Statistical Computing). There are no firm rules as to how large train or test sets should be, but we chose a 70/30 divide to minimize overfitting.^22^ There was slight variation in numbers in each sweep due to small differences in missing risk factor or concurrent correlate data, which will be reported alongside the results.

### Measures

The SDQ is a validated 25-item screening measure of children's (ages 3−16 years) mental health and behavioral problems that is used in both clinical and research settings.^20^ In this study, caregiver-completed SDQ data were collected at ages 5, 7, 11, and 14 years—sweeps 3, 4, 5, and 6, respectively. The MFQ, a reliable and validated self-report measure of depressive feelings and behaviors in children and adolescents (ages 6−17 years),^21^ was completed at age 14 years. Finally, the item “In the past year have you hurt yourself on purpose?” which was administered at age 14 years, was used as an indicator of self-harm. Brief items assessing self-harm have been widely used across studies^3^^,^^12^^,^^13^ and large national surveys.^23^ Prior research has also indicated that YPSH in community-based samples are more likely to accurately respond to these items because of their less triggering and invasive nature.^24^ The SDQ, MFQ, and self-harm item were used in data-driven analyses to describe the profile(s) of YPSH.

As self-harm risk is a nuanced phenomenon with multiple types of risk factors that could vary according to emotional and behavioral profiles, data-driven approaches—specifically, machine learning algorithms—are ideal for assessing complex relationships among a large number of possible risk factors in a replicable manner.^7^ For this study, a large number (75−97 per sweep) of potential risk factors or concurrent correlates were selected from the MCS dataset on the basis of previous literature (Supplement 1, available online) and more general developmental factors that are associated with a host of different behavioral and mental health outcomes.^25^ We grouped these variables according to 6 domains that have been broadly discussed across prior self-harm research and theoretical models: child health (eg, sleep, alcohol consumption)^18^; child mental health (eg, emotional issues, self-esteem)^5^^,^^9^; caregiver mental health (eg, health limitations)^9^^,^^14^; home environment (eg, housing tenure, neighborhood safety)^26^^,^^27^; peer relations (eg, quality of friendships)^10^^,^^11^; and adversity (eg, bullying).^10^

### Statistical Analysis

Our first goal was to characterize the profiles of YPSH at age 14 years. To do this, we entered *z*-scored SDQ and MFQ data to a simple artificial neural network^28^ using the neural network toolbox in Matlab 2018a (MATLAB and Statistics Toolbox Release). This is an unsupervised machine learning algorithm that learned about different profiles of scores across measures in the N = 10,827 dataset. This type of nonlinear data reduction technique is ideally suited to high-dimensional datasets because, unlike other data reduction techniques, it does not group variables or identify latent factors. Instead, it preserves information about potentially distinct profiles within the dataset, captures nonlinear relationships, and allows for measures to be differentially related across the sample.^29^, ^30^, ^31^ We then used k-means clustering to determine whether different subgroups of YPSH existed and how members of those subgroups differed from each other.

To identify longitudinal risk factors and concurrent correlates for subgroup membership, we used logistic regression with regularization through Least Absolute Shrinkage and Selection Operator (LASSO)^32^ on our train samples in Matlab. LASSO is a supervised algorithm typically used for feature selection regularization. It models the outcome (ie, self-harm subgroup versus comparison) while emphasizing the most important variables by shrinking coefficients of less important variables to zero. For this second analysis, participants missing more than 30% of the risk factor or concurrent correlate variables were excluded, whereas those with less than 30% missing data had missing values imputed through k-nearest-neighbor (KNN) imputation (k = 25).^33^ This is important because, unlike our first analysis, this second analysis would use almost a decade of preceding data. Excluding any participant with 1 or more missing value could result in a heavily biased sample. In reality, the overall amount of imputed data was very small, only 0.95% to 1.96% depending upon the sweep (see Figures S1−S5 and Supplement 2, available online). We elected to use KNN imputation because it performs equally well as, if not better than, the more complex imputation approaches, and is also more suitable for continuous data than alternatives such as multivariate imputation by chained equations.^34^

Within the train sample, each sweep was subjected to 1,000 bootstrapping iterations of logistic regression with LASSO regularization and 5-fold cross-validation. As this procedure selects stronger variables while down-weighting weaker variables within each iteration, we selected only those variables that were non-zero in 95% of iterations. Finally, to verify their predictive accuracy, the variables selected using this cross-validation within the training sample were used to predict self-harm in the test sample through standard logistic regression. Only the variables that survived all of these steps, including the final validation on the test samples, were considered genuine risk factors or concurrent correlates of self-harm behavior.

## Results

Our artificial neural network and clustering analysis on the resulting node weights from the SDQ subdomain and MFQ total scores identified 2 clusters of YPSH: 1 cluster with high SDQ and MFQ scores reflecting psychopathology (n = 379, labeled “Group 1”), and 1 cluster with more age-appropriate scores reflecting lack of psychopathology (n = 905, labeled “Group 2”) (Table 1). The silhouette coefficient, a validity measure for cluster analyses, was 0.52 for this solution. This indicates good separation between the 2 subgroups of YPSH.^35^ Table 2 shows descriptive information for all group samples.

**TABLE 1:** Means and SDs of Strength and Difficulties Questionnaire (SDQ) Total Scores and Mood and Feelings Questionnaire (MFQ) Total Scores for Each Subgroup of Young People Who Self-Harm and the Comparison Sample at Age 14 Years

	SDQ total score Mean (SD)	MFQ total score Mean (SD)
**Group**		
Group 1	17.67 (5.10)	15.14 (6.72)
Group 2	6.41 (3.56)	12.03 (6.87)
Comparison	6.92 (5.22)	4.41 (4.43)

**TABLE 2:** Descriptive Information for the Complete Case Samples of Each Group

	Group 1 (n = 379)	Group 2 (n = 905)	Comparison (n = 900)
Sex (%)			
Female	269 (71)	686 (76)	630 (70)
Male	110 (29)	219 (24)	270 (30)
Ethnicity (%)			
White	349 (92)	822 (91)	790 (88)
Mixed	11 (3)	29 (3)	18 (2)
Indian	3 (1)	13 (1)	19 (2)
Pakistani and Bangladeshi	8 (2)	21 (3)	44 (5)
Black or Black British	5 (1)	12 (1)	22 (2)
Other ethnic group	3 (1)	8 (1)	7 (1)
OECD below 60% median poverty (%)			
Age 5 y	179 (47)	217 (24)	206 (23)
Age 7 y	157 (41)	179 (20)	197 (22)
Age 11 y	127 (34)	125 (14)	145 (16)
Age 14 y	148 (39)	186 (21)	177 (20)

Note: Data are n (%). Numbers vary because of missing data. OECD = organization of economic co-operation and development.

**FIGURE 1:**
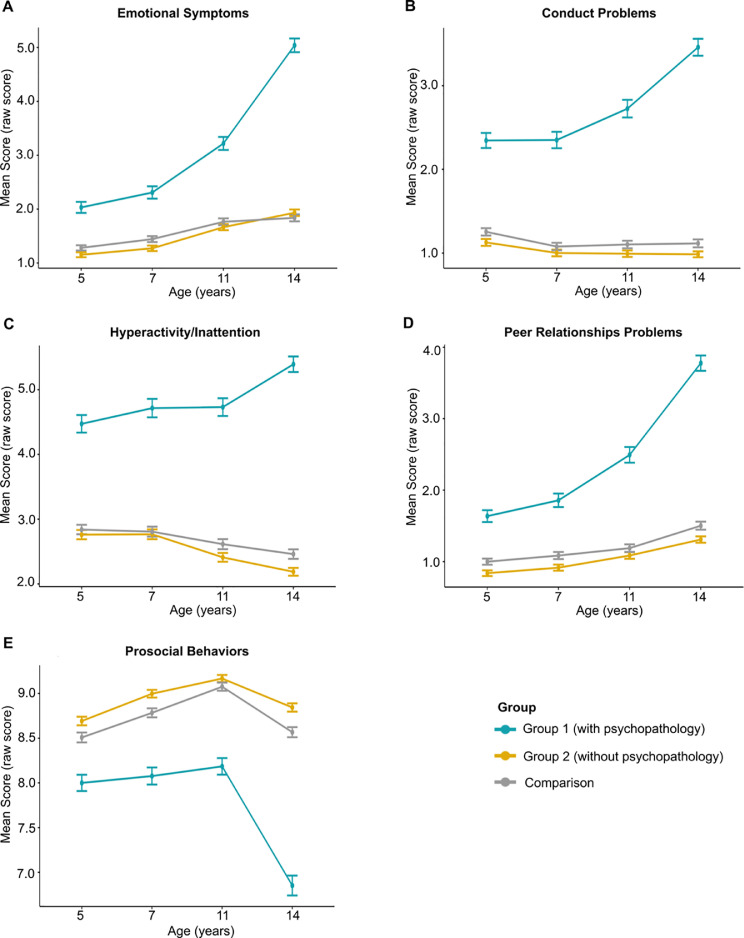
Developmental Trajectories From Ages 5 to 14 for Group 1, Group 2, and the Comparison Group of Mean Strength and Difficulties Questionnaire (SDQ) Subdomain Raw Scores ***Note:****Error bars show the 95% CIs.*

We initially tracked the SDQ scores back through earlier sweeps, to test whether these differences were consistent across developmental time (Figure 1). A 2-factor mixed analysis of variance, Greenhouse−Geisser corrected, showed a significant interaction between group membership (Group 1 [with psychopathology], Group 2 [without psychopathology], and Comparison) and age (ages 5, 7, 11, and 14 years) for all SDQ subdomain scores (Emotional symptoms: *F*_5.52,6020.70_ = 96.20, *p* < .0001; Conduct problems: *F*_5.64,6149.33_ = 47.23, *p* < .0001; Hyperactivity/Inattention: *F*_6.65,6059.84_ = 27.92, *p* < .0001; Peer relationship problems: *F*_5.55,6054.50_ = 66.44, *p* < .0001; Prosocial behaviors: *F_5.48,5980.45_* = 33.67, *p* < .0001). These interactions result from the increasing psychopathology for Group 1 and their increasing divergence from the other 2 groups growing over developmental time (versus Group 2: all *F* values >51.64, all *p* values <.0001; versus Comparison: all *F* values >36.78, all *p* values <.05), whereas the trajectories for the other 2 groups did not differ except for emotional symptoms (Group 2 versus Comparison: Emotional symptoms (*F*_2.77,4989.98_ = 3.680), *p* = .014; all other *F* values >0.31, all other *p* values >.05).

**FIGURE 2:**
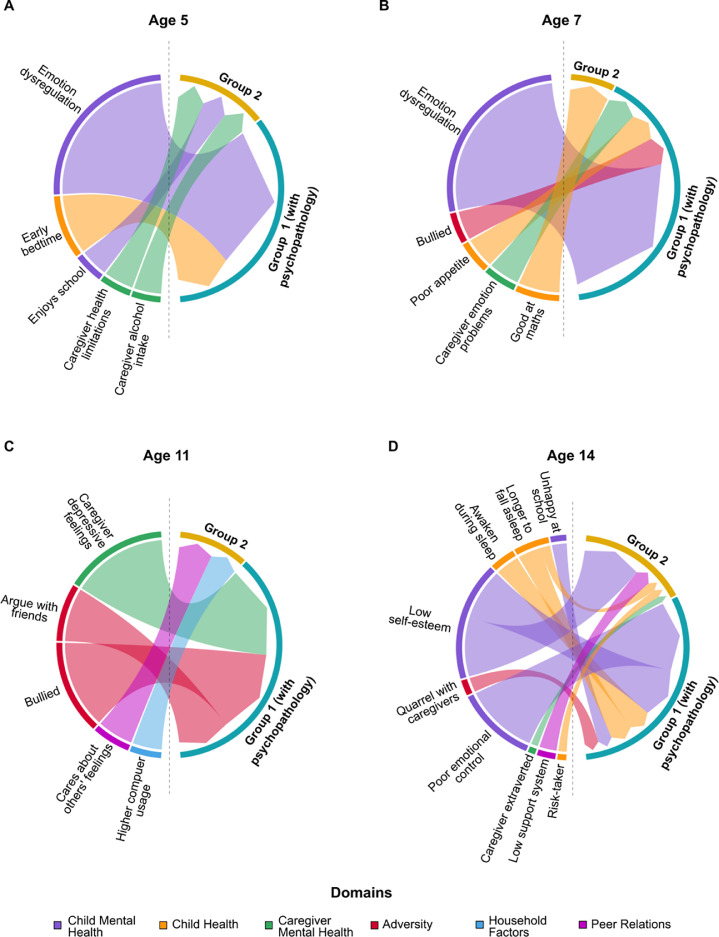
Risk Factors and Concurrent Correlates of Self-Harm for Group 1 (With Psychopathology) and Group 2 Membership (Without Psychopathology) From Ages 5 to 14 Years ***Note:****Risk factors and concurrent correlates across 6 domains from (A) Age 5 years, (B) Age 7 years, (C) Age 11 years, and (D) Age 14 years. The size of each arc is proportional to the β value, and thus the strength, of each risk factor.*

After applying the cross-validated LASSO on the train set and then testing the surviving factors in the test set, we identified concurrent self-harm correlates at age 14 years, when participants answered the self-harm indicator, of subgroup membership (Figure 2D). The strengths of the validated correlates for both groups were assessed by the absolute values of the standardized coefficients computed from the LASSO procedure. The strongest correlates for Group 1 (with psychopathology; n = 249, 73% female) were poor emotional control (β = 0.70), low self-esteem (β = 0.50), waking during sleep (β = 0.22), trouble falling asleep (β = 0.21), more quarrels with caregivers (β = 0.14), and being unhappy at school (β = 0.14). Correlates for Group 2 (without psychopathology; n = 614, 77% female) were low self-esteem (β = 0.57), low support system from peers/family (β = 0.16), trouble falling asleep (β = 0.09), being more willing to take risks (β = 0.08), and having caregivers with self-reported higher extraversion (β = 0.07).

Longitudinal risk factors were identified at ages 5, 7, and 11 years (Figure 2A−C). At age 11 years, later membership of Group 1 (with psychopathology; n = 251, 73% female) was predicted by caregiver depressive feelings (β = 0.26, *p* = .052), being bullied (β = 0.24), and frequently arguing with friends (β = 0.15). Later membership of Group 2 (n = 608, 76% female) was predicted by caring about the feelings of others (β = 0.10) and more weekday hours spent on the computer/games (β = 0.08). At age 7 years, the risk factors for Group 1 (n = 271, 73% female) were child emotion dysregulation issues (β = 0.40), poorer appetite (β = 0.07), caregiver emotional problems (β = 0.07), and being bullied (β = 0.07). Group 2 membership (n = 644, 76% female) was predicted by having less difficulty with mathematics (β = 0.09) at age 7 years. Finally, at age 5 years, risk factors of later Group 1 membership (n = 271, 73% female) were child emotion dysregulation issues (β = 0.28) and earlier weekday bedtimes (β = 0.10), whereas later Group 2 membership (n = 644, 76% female) was predicted by caregiver health limitations on work (β = 0.05), child enjoying school (β = 0.05), and more frequent caregiver alcohol intake (β = 0.05).

In summary, we identified distinct subgroups among YPSH, with significant risk factors present as early as age 5 years, nearly a decade before these individuals reported self-harming. Although sleep difficulties and low self-esteem reported at age 14 years were commonly associated with self-harm behavior, irrespective of subgroup, there were divergences in other risk factors. The longitudinal nature of our analysis allowed us to distinguish factors that appear alongside reported self-harm behavior (eg, low self-esteem) from those that precede it (eg, bullying). The pathway for Group 1 YPSH embodies a “psychopathology” route, in which a long history of emotional dysregulation, psychopathology, and bullying precedes self-harm. Members of Group 2, meanwhile, do not fit this profile or that suggested by previous research.^13^ Their self-harm behavior is harder to predict early in childhood, and instead coincides with later increases in risk-taking and changes in their relationships with family and friends—the “adolescent risky behavior” pathway.

## Discussion

Our finding of 2 distinct subgroups of YPSH, based on emotional, behavioral, and mental health measures, in a nationally representative cohort further supports the notion that YPSH is not one homogenous group. This is especially important, as most self-harm models are based upon clinical samples.^36^ Although models vary (eg, Affect Regulation Model,^36^ Experiential Avoidance Model [EAM]^5^), they broadly conceptualize self-harm as a negative behavior that provides a form of escape, management, or regulation of unwanted emotions.^5^ The expected profile of YPSH based on these models would be individuals with depressive symptoms, low self-esteem, interpersonal/familial challenges, early adversity, and environmental stressors^5,36^—risk factors that are most commonly assessed in empirical studies on self-harm. YPSH in Group 1 (with psychopathology) appear to match this profile, as they were reported by their caregivers to have emotional and behavioral difficulties as early as age 5 years, which gradually deteriorated over time. They also self-reported poor mental health at age 14 years when they reported self-harm. Unexpectedly, however, we found a much larger subgroup of YPSH (Group 2) who do not present the psychopathological traits that have been most associated with those who self-harm, unlike their Group 1 counterparts. This group fits the nonpsychopathological profile reported by Stanford *et al.* and underscores the value of the “profile approach” in being able to assess multiple profiles and potentially novel risk factors for adolescent self-harm.^13^ Group 2 YPSH are indeed distinct from the comparison sample, as our subsequent analysis of concurrent and preceding risk factors would reveal.

Child Mental Health, Adversity, and Caregiver Mental Health domains, which are heavily emphasized across the self-harm literature,^5^^,^^10^ were particularly significant for YPSH in Group 1 (with psychopathology). In fact, the strongest risk factor both at ages 5 and 7 years for Group 1 membership was emotion dysregulation, while concurrent correlates included poor emotional control and low self-esteem. From a theoretical standpoint, having persistent emotional problems puts one at high risk for various forms of psychopathology^25^ and is postulated as the root of future self-harm in models such as EAM.^5^ In addition, adversity in the form of bullying was a strong, early risk factor, which is thought to exacerbate mental health and adjustment difficulties, even after the bullying has stopped.^37^ Furthermore, caregivers of Group 1 YPSH faced poorer mental health throughout development, unlike those of Group 2—a risk factor for both self-harming behavior^9^ and developmental challenges.^25^ The YPSH in Group 1 appear to face persistent internal and external challenges across development that may increase risk of self-harm on a pathway reflecting developmental psychopathology^25^: a path that starts early, with its origins in adverse experiences of bullying and poor mental health for both children and their caregivers. Although sharing the concurrent correlate of low self-esteem, Group 2 (without psychopathology) did not present any negative risk factors across these domains. By neither fitting the profile nor having the risk factors expected from theoretical underpinnings, this group of YPSH reinforces the necessity of a more multidimensional assessment of self-harm risk.

Machine learning approaches for complex classification outcomes such as self-harm provide a way to gauge more nuanced risk factors, particularly for unexpected profiles.^16^ Although there was a lack of early and consistent risk factors, the key domains for the adolescent risky behavior pathway of the larger nonpsychopathological Group 2 of YPSH were Peer Relations and Child Health. In particular, being more willing to take risks was a significant correlate of this subgroup. Risk taking has been empirically and conceptually linked to self-harm, as both are subject to peer influence^38^ and impulsivity^39^^,^^40^: factors that may limit time spent considering alternative coping methods and the consequences of self-harm.^13^ Peer relations factors were also important for this subgroup, including a low support system as a concurrent correlate but also a greater concern about the feelings of others as a risk factor at age 11 years. Lack of social support and peer-related problems have been significant risk factors in longitudinal studies investigating self-harm.^41^ Interestingly, at ages 5 and 7 years, caregivers reported positive school-related risk factors, but neither was particularly strong. Hypothetically, these YPSH do not externalize their difficulties, especially as they do not seem to feel safe with their family/friends. They may also find it difficult to connect with their caregivers’ extraversion, a concurrent correlate, who therefore are unaware of their struggles—which may explain the case of why many self-harm incidents are unknown by caregivers.^42^

A key implication of our findings is that we have a decade-long window to intervene for some children who are at increased risk for self-harm as adolescents. Early targeted interventions, particularly those focused on emotion regulation, may be helpful for this group. A meta-analysis on resilience interventions in schools highlights that effectiveness can depend on age and mental health outcomes; for instance, childhood interventions are relatively effective for general psychological distress.^43^ The persistence of psychopathology among Group 1 YPSH, furthermore, suggests that early screening measures if combined with prompt access to effective intervention could reduce the risk of future self-harm as well as improve mental health in the short term.^8^ A second and highly tractable target for intervention is bullying, which casts a shadow over adult as well as childhood mental health.^37^ This was a strong and early risk factor of self-harm for children in the psychopathology pathway, preceding self-harm reports by 7 years. There are now a number of evidence-based anti-bullying interventions that can be deployed at a school level that could, and should, be implemented.^44^^,^^45^

The larger Group 2 (without psychopathology) represents the challenge that we face to assist those individuals in the general population.^13^ However, their indication of poorer mental health on the MFQ than that of the comparison sample at age 14 years, as well as both risk taking and peer-related factors, suggest that access to universal programs and materials for self-help and problem-solving/conflict regulations (as recommended for inclusion in Personal, social, health and economic [PSHE] education^46^) may be effective—especially for those who do not seek help from formal services. Adolescent mental health screening measures or clinical interviews, furthermore, should include self-harm items with careful consideration of non-stigmatizing language.^8^^,^^13^ Sleep training is also an area to consider. Sleep difficulties were strong overlapping concurrent correlates for self-harming behavior in our study, and have been associated with emotion regulation and mood disorders^47^ as well as increased suicide risk.^18^ It would be worthwhile to further investigate whether these sleep troubles occur earlier on in development, along with possible treatments. In addition, targeted interventions by mental health leaders and school-based mental health teams are important. Training for teachers, especially, could be critical, as they are often the first people to hear about self-harm but may have difficulty responding. This training for education faculties alongside the repurposing of evidence-based interventions, like those for anti-bullying,^45^ to enhance support systems and peer relationships from an earlier stage may provide more preventive and non-stigmatizing measures.

As there is a lack of research investigating early childhood origins of self-harm^8^^,^^10^ and subgroups among YPSH, our study provides an important foundation for future research. An essential future direction is to replicate our findings in other national and international cohorts, particularly as our work only partially replicated that of previous groups.^13^^,^^15^ Thus, a next step is to further refine psychological subgroups and risk factors of YPSH. In addition, where anti-bullying programs are trialed or implemented, permission to link to administrative data (eg, health records) would allow future research to explore presentations for self-harm among the cohort in adolescents. This may help build a stronger evidence base for community-based interventions, which could be informative for clinical interventions as well.^8^ Targeted prevention for substance misuse has successfully applied personality measures to tailor school-based programs to pupils’ needs; our findings suggest that a similar approach may be worth considering among pre-teens in relation to self-harm.^48^

The longitudinal analysis of a nationally representative sample, along with powerful predictive analyses using machine learning, provides valuable insight into the developmental pathways leading to self-harm. Nevertheless, several limitations to this study exist. First, the self-harm indicator used in this study is a binary yes-or-no response, despite the complex nature and range in severity of self-harm.^5^ The way in which participants hurt themselves or more probing questions into the motivations behind this behavior were not collected. However, a recent report suggests that a brief yes/no item assessing self-harm is particularly advantageous for large-scale studies, as it is likely to be less triggering and invasive, which may motivate more open responses from both male and female YPSH.^24^ Furthermore, the self-harm rate that we found in our study (approximately 14.6%) matches what has been reported from prior studies in community cohorts.^3^ Despite not capturing all nuances, the simple measure of asking whether children have self-harmed in the previous year still provided a concrete window into a mental health problem that is extremely difficult to measure accurately and in detail, particularly given the large sample size. Second, we intentionally did not include the sex of the participants as a risk factor. Instead, we incorporated the fact that approximately 70% to 77% of the YPSH were female participants in our matched comparison sample. This pattern is well-established,^10^ and, even in a population sample, separate analyses by sex would suffer from low statistical power from which to explore self-harm among male participants. Moreover, as a UK representative cohort, our sample is over 90% White, which broadly reflects governmental statistics indicating that 87% of people in the United Kingdom are White.^49^ This limits our capability to investigate potential intersectionality between ethnicity and self-harm risk profiles, and highlights the importance of replicating our study with other nationally representative samples. Finally, our statistical approach was incredibly conservative (cross-validated regularization, bootstrapping, and a final validation in a test sample) when analyzing the risk factors for this outcome, which may serve as both a strength and limitation of this study. It is likely that we overlooked meaningful weaker risk factors, but this comes at the benefit of avoiding overfitting and suggests that our reported findings are robust.

There is global consensus that self-harm is a prevalent concern in adolescence and a priority for public health efforts. Establishing early risk factors and profiles that can be traced and tracked across development provides a crucial step toward the early identification of these young people, to elucidating underlying casual mechanisms and, ultimately, prevention and treatment. We show that there are 2 relatively distinct profiles among adolescents who self-harm—early and persistent psychopathology and exposure to bullying versus adolescent peer relations challenges and risk taking—and that these profiles have different developmental pathways.

## Appendix Supplementary Materials

Image, application 1
