# Supplementary material for: Two Pathways to Self-Harm in Adolescence
Source: J Am Acad Child Adolesc Psychiatry. 2021 Dec;60(12):1491–500. doi: 10.1016/j.jaac.2021.03.010 (PMC8661039; doi:10.1016/j.jaac.2021.03.010)
Supplement: Supplementary file 1 [file mmc1.docx]

**Supplement 1: List of all variables per sweep used for LASSO model to identify predictors.**

| **Sweep 3 Variables: Age 5** |  |
| --- | --- |
| **Variable** | **Domain** |
| How often CM spends time with friends outside school | Relations (Peer) |
| Closeness to CM | Relations |
| Caregivers married | Household Factors |
| Caregivers cohabiting | Household Factors |
| Caregivers neither married nor cohabiting | Household Factors |
| Natural mother resident full-time in household | Household Factors |
| Natural mother resident part-time in household | Household Factors |
| Natural mother deceased | Household Factors |
| Natural mother non-resident, in contact | Household Factors |
| Natural mother non-resident, not in contact | Household Factors |
| Natural mother non-resident, contact not known | Household Factors |
| Natural father resident full-time in household | Household Factors |
| Natural father resident part-time in household | Household Factors |
| Natural father deceased | Household Factors |
| Natural father non-resident, in contact | Household Factors |
| Natural father non-resident, not in contact | Household Factors |
| Natural father non-resident, contact not known | Household Factors |
| Number of siblings in household | Household Factors |
| OECD Score (derived) | Household Factors |
| OECD equivalised income (derived) | Household Factors |
| OECD below 60% median poverty indicator | Household Factors |
| Natural mother's BMI at interview | Household Factors |
| Respondent NVQ highest level across sweeps 1,2, 3 | Household Factors |
| Caregiver in work | Household Factors |
| Hours per term-time weekday watching tv/dvd | Household Factors |
| Hours per term-time weekday playing on computer | Household Factors |
| Satisfaction with education at current school | Household Factors |
| Usually has midday meal provided by school | Household Factors |
| Caregiver receiving state benefits/payments | Household Factors |
| Caregiver receives child support/maintenance payments | Household Factors |
| Caregiver saves regularly | Household Factors |
| How well managing financially | Household Factors |
| Home is really disorganised | Household Factors |
| Cannot hear yourself think at home | Household Factors |
| Atmosphere is calm at home | Household Factors |
| Good area for raising children | Household Factors |
| Friends live in the area | Household Factors |
| Family live in the area | Household Factors |
| Friends and family live in the area | Household Factors |
| No friends/family live in the area | Household Factors |
| How safe they fell in the area | Household Factors |
| None NVQ | Household Factors |
| Own outright, mortgage/loan, part rent/part mortgage | Household Factors |
| Rent from local authority, rent from housing association, living with parents, rent free, other | Household Factors |
| Privately rent | Household Factors |
| Both caregivers employed | Household Factors |
| One caregiver employed | Household Factors |
| Caregivers unemployed | Household Factors |
| Child Social Behaviour Questionnaire (Independence-Self-Regulation) | Child Mental Health |
| Child Social Behaviour Questionnaire (Emotional-Dysregulation) | Child Mental Health |
| CM bothered by emotional problems | Child Mental Health |
| Days per week CM does sport/exercise | Child Health |
| Bedtime on weekdays (term-time) | Child Health |
| CM sleeping habits a problem | Child Health |
| CM eats at regular times | Child Health |
| No concerns about CM speech/language | Child Health |
| CM language developing slowly | Child Health |
| CM doesn't seem to understand other people | Child Health |
| CM pronounces words poorly | Child Health |
| CM doesn't hear well | Child Health |
| CM stutters | Child Health |
| CM has other speech/language concerns | Child Health |
| CM enjoys school | Child Health |
| CM reluctant to go to school | Child Health |
| CM's general level of health | Child Health |
| CM has longstanding illness | Child Health |
| How often CM wets bed at night | Child Health |
| Days per week CM eats breakfast | Child Health |
| Portions of fruit per day | Child Health |
| Concern for CM becoming overweight | Child Health |
| CM taking regular medication | Child Health |
| Anyone smokes near CM | Child Health |
| CM BMI | Child Health |
| CM Obesity flag | Child Health |
| Caregiver Kessler (K6) Scale | Caregiver Health |
| Caregiver's general health | Caregiver Health |
| Caregiver's health limits physical activities | Caregiver Health |
| Caregiver's health limits work/study | Caregiver Health |
| Caregiver has longstanding illness | Caregiver Health |
| How often caregiver drinks alcohol | Caregiver Health |
| Parenting competence | Caregiver Health |
| Caregiver used recreational drugs in the past 12 months | Caregiver Health |
| Caregiver satisfied with partner's work/family balance | Caregiver Health |
| Caregiver satisfied with own life so far | Caregiver Health |
| How often caregiver and partner disagree re CM issues | Adversity |

| **Sweep 4 Variables: Age 7** |  |
| --- | --- |
| **Variable** | **Domain** |
| CM: number of friends | Relations (Peer) |
| CM has best friends | Relations (Peer) |
| CM enjoys playing with friends | Relations (Peer) |
| CM: how well get along with siblings | Relations (Peer) |
| CM: how often have fun with family on weekends | Relations (Peer) |
| How often CM spends time with friends outside school | Relations (Peer) |
| How close caregiver is to CM? | Relations |
| How often caregiver enjoys listening and doing things with CM | Relations |
| How often caregiver expresses affection by hugging, kissing CM | Relations |
| How often caregiver gets irritated with CM | Relations |
| Caregivers married | Household Factors |
| Caregivers cohabiting | Household Factors |
| Caregivers neither married nor cohabiting | Household Factors |
| Natural mother resident full-time in household | Household Factors |
| Natural mother resident part-time in household | Household Factors |
| Natural mother deceased | Household Factors |
| Natural mother non-resident, in contact | Household Factors |
| Natural mother non-resident, not in contact | Household Factors |
| Natural mother non-resident, contact not known | Household Factors |
| Natural father resident full-time in household | Household Factors |
| Natural father resident part-time in household | Household Factors |
| Natural father deceased | Household Factors |
| Natural father non-resident, in contact | Household Factors |
| Natural father non-resident, not in contact | Household Factors |
| Natural father non-resident, contact not known | Household Factors |
| Number of siblings in household | Household Factors |
| Time (months) at current address | Household Factors |
| OECD equivalised income (derived) | Household Factors |
| OECD below 60% median poverty indicator | Household Factors |
| OECD income weighted quintiles (UK analysis) | Household Factors |
| Caregiver in work | Household Factors |
| Caregiver NVQ Highest Level (all sweeps) | Household Factors |
| Same address as last interview | Household Factors |
| Caregiver satisfaction with education at current school | Household Factors |
| Hours per weekday CM watches TV or videos | Household Factors |
| CM usually has midday meal provided by school | Household Factors |
| Caregiver receives any benefits | Household Factors |
| Caregiver receives child support/maintenance payments | Household Factors |
| Caregiver no NVQ | Household Factors |
| Home: own outright, mortgage/loan, part rent/part mortgage | Household Factors |
| Home: rent from local authority/housing association, living with parents, rent free, other | Household Factors |
| Home: privately rent | Household Factors |
| Both caregivers employed | Household Factors |
| One caregiver employed | Household Factors |
| Caregivers unemployed | Household Factors |
| Child Social Behaviour Questionnaire (Independence-Self-Regulation) | Child Mental Health |
| Child Social Behaviour Questionnaire (Emotional-Dysregulation) | Child Mental Health |
| Child Social Behaviour Questionnaire (Cooperation) | Child Mental Health |
| CM: how often feels happy | Child Mental Health |
| CM: how often worried | Child Mental Health |
| CM: how often feels sad | Child Mental Health |
| CM: how often are quiet | Child Mental Health |
| CM: how often like to be in school | Child Mental Health |
| CM: how often laughs | Child Mental Health |
| CM: how often lose temper | Child Mental Health |
| CM: how much likes school | Child Mental Health |
| CM: how often feels unhappy at school | Child Mental Health |
| CM enjoys school | Child Mental Health |
| How often CM reluctant to go to school | Child Mental Health |
| CM has difficulty at school with maths | Child Health |
| CM has difficulty at school with reading | Child Health |
| CM has difficulty at school with writing | Child Health |
| CM has difficulty at school with Phys Ed | Child Health |
| Days per week CM does sport/exercise | Child Health |
| CM's physical activity frequency | Child Health |
| Frequency of CM's physical activities with family | Child Health |
| CM's regular bedtime on term-time weekdays | Child Health |
| Frequency caregiver takes child to park/playground | Child Health |
| How often CM wets bed at night | Child Health |
| CM's general level of health | Child Health |
| Whether CM has longstanding illness | Child Health |
| Days per week CM eats breakfast | Child Health |
| CM has a variety of foods | Child Health |
| CM's appettite | Child Health |
| Anyone smokes near CM | Child Health |
| Natural mother BMI at interview | Caregiver Health |
| Caregiver Neuroticism subscale (OCEAN) | Caregiver Health |
| Caregiver Extrovert subscale (OCEAN) | Caregiver Health |
| Caregiver Kessler (K6) Scale | Caregiver Health |
| Caregiver's general health | Caregiver Health |
| Caregiver Mental Health limits work/study etc | Caregiver Health |
| Caregiver's emotional problems limit work/study etc | Caregiver Health |
| Frequency caregiver consumes alcohol | Caregiver Health |
| CM: how often bullied by other children | Adversity |
| CM: how often feel left out | Adversity |
| CM bullied at school | Adversity |

| **Sweep 5 Variables: Age 11** |  |
| --- | --- |
|  |  |
| **Variable** | **Domain** |
| Friends live in the area | Relations (Peer) |
| Friends and family live in the area | Relations (Peer) |
| No friends/family live in the area | Relations (Peer) |
| Amount of time caregiver spends with child | Relations (Peer) |
| How often CM spends time with friends outside school | Relations (Peer) |
| CM: how often do exchange messages with friends on the internet | Relations (Peer) |
| CM: how often visit a social networking website on the internet | Relations (Peer) |
| CM: how many friends live in the same area | Relations (Peer) |
| CM: how often spend time with your friends outside of school | Relations (Peer) |
| How many CM's friends smoke cigarettes | Relations (Peer) |
| How many CM's friends drink alcohol | Relations (Peer) |
| CM doesn't know if friends smoke | Relations (Peer) |
| CM doesn't know if friends drink alcohol | Relations (Peer) |
| How close caregiver is to CM | Relations |
| Caregiver NVQ Highest Level (all sweeps) | Household Factors |
| Caregivers married | Household Factors |
| Caregivers cohabiting | Household Factors |
| Natural mother resident full-time in household | Household Factors |
| Natural mother resident part-time in household | Household Factors |
| Natural mother non-resident, not in contact | Household Factors |
| Natural mother non-resident, contact not known | Household Factors |
| Natural father resident full-time in household | Household Factors |
| Natural father resident part-time in household | Household Factors |
| Natural father deceased | Household Factors |
| Natural father non-resident, in contact | Household Factors |
| Natural father non-resident, not in contact | Household Factors |
| Natural father non-resident, contact not known | Household Factors |
| Number of siblings in household | Household Factors |
| OECD equivalised weekly family income | Household Factors |
| OECD below 60% median poverty indicator | Household Factors |
| OECD equivalised income quintiles - UK whole | Household Factors |
| OECD equivalised income quintiles - by country | Household Factors |
| Caregiver legally separated | Household Factors |
| Caregiver married, 1st and only marriage | Household Factors |
| Caregiver remarried, 2nd or later marriage | Household Factors |
| Caregiver single, never married | Household Factors |
| Caregiver divorced | Household Factors |
| Caregiver widowed | Household Factors |
| Caregiver has a civil partner (legally recognised) | Household Factors |
| Caregiver's general health | Household Factors |
| Receives Child Benefit payment | Household Factors |
| Managing well financially | Household Factors |
| How often anyone at home help with CM's homework | Household Factors |
| How many times family has been on holiday outside UK | Household Factors |
| Hours per weekday CM spends on computer/games | Household Factors |
| CM usually has midday meal provided by school | Household Factors |
| CM has a bedroom of their own | Household Factors |
| CM: friends' families richer | Household Factors |
| CM: friends' families poorer | Household Factors |
| CM: friends' families about the same | Household Factors |
| CM: don't know if friends' families are richer/poorer | Household Factors |
| CM: wishes family could afford to buy more of what CM wants | Household Factors |
| CM: bothered by friends having things CM doesn't | Household Factors |
| CM: safe to walk, play or hang out in the area during the day | Household Factors |
| CM: how often caregivers take an interest in CM's school work | Household Factors |
| Caregiver no NVQ | Household Factors |
| Home: own outright, mortgage/loan, part rent/part mortgage | Household Factors |
| Home: rent from local authority/housing association, living with parents, rent free, other | Household Factors |
| Home: privately rent | Household Factors |
| Both caregivers employed | Household Factors |
| One caregiver employed | Household Factors |
| Caregivers unemployed | Household Factors |
| Family live in the area | Familial Relations |
| In the last 4 weeks, how often CM felt happy | Child Mental Health |
| In the last 4 weeks, how often CM worried about what would happen | Child Mental Health |
| In the last 4 weeks, how often CM felt sad | Child Mental Health |
| In the last 4 weeks, how often CM felt afraid or scared | Child Mental Health |
| In the last 4 weeks, how often CM laughed | Child Mental Health |
| In the last 4 weeks, how often CM got angry | Child Mental Health |
| CM cares about doing well at school | Child Mental Health |
| CM feels bad/guilty when they have done something wrong | Child Mental Health |
| CM does not show my emotions to others | Child Mental Health |
| CM concerned about the feelings of others | Child Mental Health |
| CM's well-being | Child Mental Health |
| CM's self-esteem (Rosenberg scale) | Child Mental Health |
| CM gets individual support in class from teacher | Child Health |
| CM gets individual support in class from a family member | Child Health |
| CM gets support in a group, small group, booster group in school | Child Health |
| Days per week CM does sport/exercise | Child Health |
| CM's regular bedtime on term-time weekdays | Child Health |
| CM’s general level of health | Child Health |
| CM has longstanding illness | Child Health |
| CM had ADHD | Child Health |
| CM diagnosed with autism/asperger's | Child Health |
| Days per week CM eats breakfast | Child Health |
| How often CM drinks artificially sweetened drinks | Child Health |
| How many portions of fruit CM eats per day | Child Health |
| CM's appetite | Child Health |
| How much CM likes class teacher | Child Health |
| Caregiver Kessler (K6) Score | Caregiver Health |
| Caregiver has longstanding illness | Caregiver Health |
| Satisfaction with work/family balance | Caregiver Health |
| Caregiver alcohol consumption frequency | Caregiver Health |
| How happy/unhappy caregiver is in current relationship | Caregiver Health |
| Satisfaction with own life so far | Caregiver Health |
| CM: how often argue or fall out with your friends | Adversity |
| How often do other CM picked on or hurt by on purpose? | Adversity |
|  |  |

| **Sweep 6 Variables: Age 14** |  |
| --- | --- |
| **Variable** | **Domain** |
| How close CM is with mother | Relations |
| How close CM is with father | Relations |
| How close caregiver is to CM | Relations |
| Amount of time caregiver spends with child | Relations |
| CM goes to youth clubs/scouts/girlguides/other organised activities | Relations (Peer) |
| Hours per week CM spends on social networking sites | Relations (Peer) |
| CM has any close friends | Relations (Peer) |
| CM: how often spend time with close friends outside of school | Relations (Peer) |
| CM has family and friends who help CM feel safe, secure and happy. | Relations (Peer) |
| CM has someone whom they would turn to for problems | Relations (Peer) |
| Caregiver NVQ Highest Level (all sweeps) | Household Factors |
| Number of siblings in household | Household Factors |
| OECD below 60% median poverty indicator | Household Factors |
| CM Obesity flag (UK90 thresholds) | Household Factors |
| How often anyone at home helps with CM's homework | Household Factors |
| How (un)likely caregiver thinks CM will attend university | Household Factors |
| CM has own bedroom | Household Factors |
| Caregiver receives child benefit payment | Household Factors |
| Caregiver managing well financially | Household Factors |
| Safe for CM to walk/play within a mile/20 min from home | Household Factors |
| Education overseas | Household Factors |
| No father/not in contact | Household Factors |
| CM has no brothers/sisters | Household Factors |
| Home: own outright, mortgage/loan, part rent/part mortgage | Household Factors |
| Home: rent from local authority/housing association, living with parents, rent free, other | Household Factors |
| Caregiver(s) unemployed | Household Factors |
| How often CM tries best at school | Child Mental Health |
| How often CM finds school interesting | Child Mental Health |
| How often CM feels unhappy at school | Child Mental Health |
| How often CM feels school is a waste of time | Child Mental Health |
| CM: when CM is worried about something, keep it to themselves | Child Mental Health |
| CM has done cannabis | Child Mental Health |
| How much CM trusts others | Child Mental Health |
| CM's self-esteem (Rosenberg scale) | Child Mental Health |
| CM's ability to control emotions | Child Mental Health |
| How willing CM is to take risks | Child Health |
| CM: good at maths (self-rating) | Child Health |
| CM: good at science (self-rating) | Child Health |
| How often CM gets tired at school | Child Health |
| How often CM smokes cigarettes | Child Health |
| CM has ever had an alcoholic drink | Child Health |
| CM has done any other illegal drug (e.g., ecstasy, cocaine, speed) | Child Health |
| Days per week CM eats breakfast | Child Health |
| How often CM eats at least 2 portions of fruit | Child Health |
| How often CM eats at least 2 portions of vegetables | Child Health |
| How often CM drinks artificially sweetened drinks | Child Health |
| How often CM eats fast food | Child Health |
| CM's general level of health | Child Health |
| During the last 4 weeks how long it takes for CM to fall asleep | Child Health |
| During the last 4 weeks how often CM awakened during sleep | Child Health |
| CM has longstanding illness | Child Health |
| CM diagnosed with autism/asperger's | Child Health |
| Caregiver Kessler (K6) Score | Caregiver Health |
| Caregiver Openness subscale (OCEAN) | Caregiver Health |
| Caregiver Conscientiousness subscale (OCEAN) | Caregiver Health |
| Caregiver Extraversion subscale (OCEAN) | Caregiver Health |
| Caregiver Agreeableness subscale (OCEAN) | Caregiver Health |
| Caregiver Neuroticism subscale (OCEAN) | Caregiver Health |
| Caregiver alcohol consumption | Caregiver Health |
| Caregiver's general health | Caregiver Health |
| Caregiver has longstanding illness | Caregiver Health |
| Caregiver ever diagnosed with depression/serious anxiety | Caregiver Health |
| Caregiver used recreational drugs in past 12 months | Caregiver Health |
| Caregiver happy with relationship with partner | Caregiver Health |
| Caregiver satisfied with life | Caregiver Health |
| How often CM argues with mother | Adversity |
| How often CM argues with father | Adversity |
| How often brothers or sisters hurt or pick on CM | Adversity |
| How often other children hurt or pick on CM | Adversity |
| How often other children bullied CM online | Adversity |
| CM insulted, threatened, shouted at | Adversity |
| Someone has been physically violent towards CM | Adversity |
| Someone has hit or used a weapon against CM | Adversity |
| Someone has stolen something from CM | Adversity |
| Someone has sexually assaulted CM | Adversity |
| Frequency CM quarrels with parent | Adversity |
|  |  |

**Supplement 2: Data Imputations**

For the feature selection analysis (LASSO) to identify self-harm risk factors from each sweep, cohort members missing more than 30% of their data (the variables listed above) were excluded, while the rest were imputed using a k nearest neighbors algorithm. Imputation is important as this prevents bias in our LASSO analyses towards those participants with more than a decade of complete data. This is important to achieve a sample that is more representative, relative to when all individuals with one or more missing values are excluded. The imputation algorithm calculates the Euclidean distance between the 25 nearest neighbors with complete data for each participant and then uses the median of these nearest neighbors to replace the participant’s missing values. Overall, the percentage of imputations done across all sweeps ranged from 0.95-1.96%. Supplementary Figures S1A-H show the proportion of subjects with missing data across each sweep while Supplementary Figures S2-S5 indicate the proportion of variables with missing data per subgroup combined with the comparison sample for all LASSO analyses.


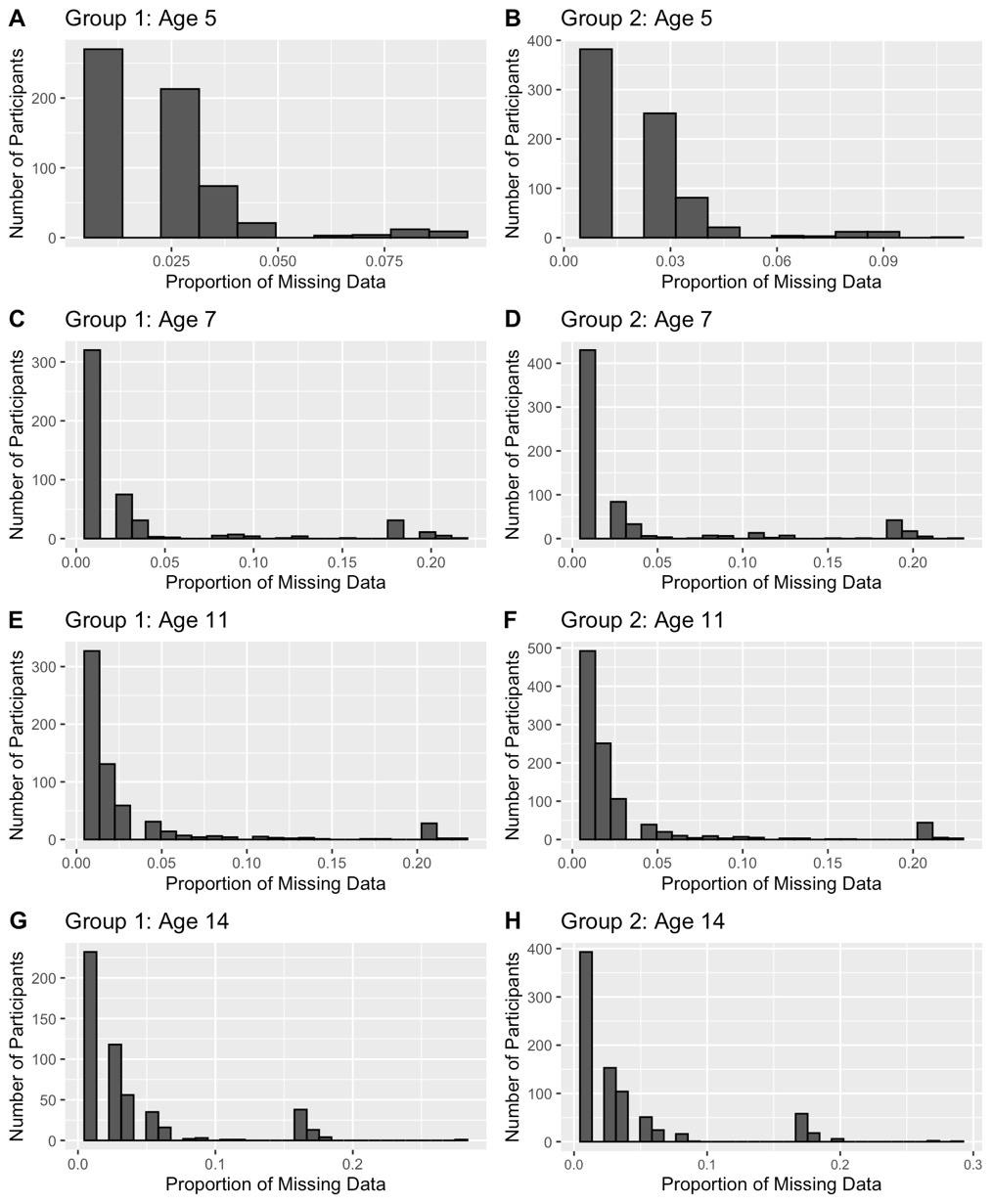


***Figures S1A-H*** *shows the proportions of subjects with missing data per subgroup (Group 1 (with psychopathology) and Group 2 (without psychopathology)) across each sweep (Age)*

**2A**


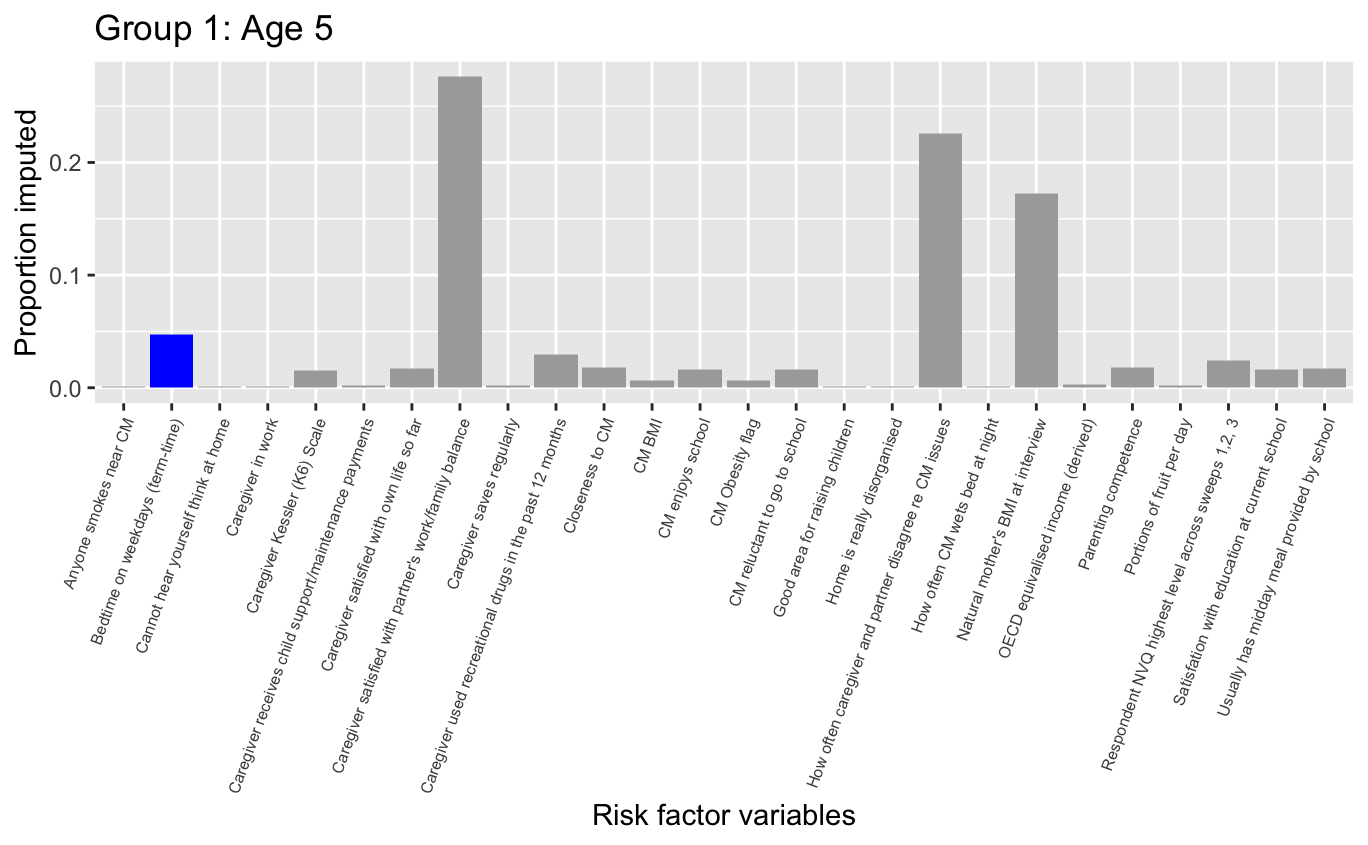


**2B**


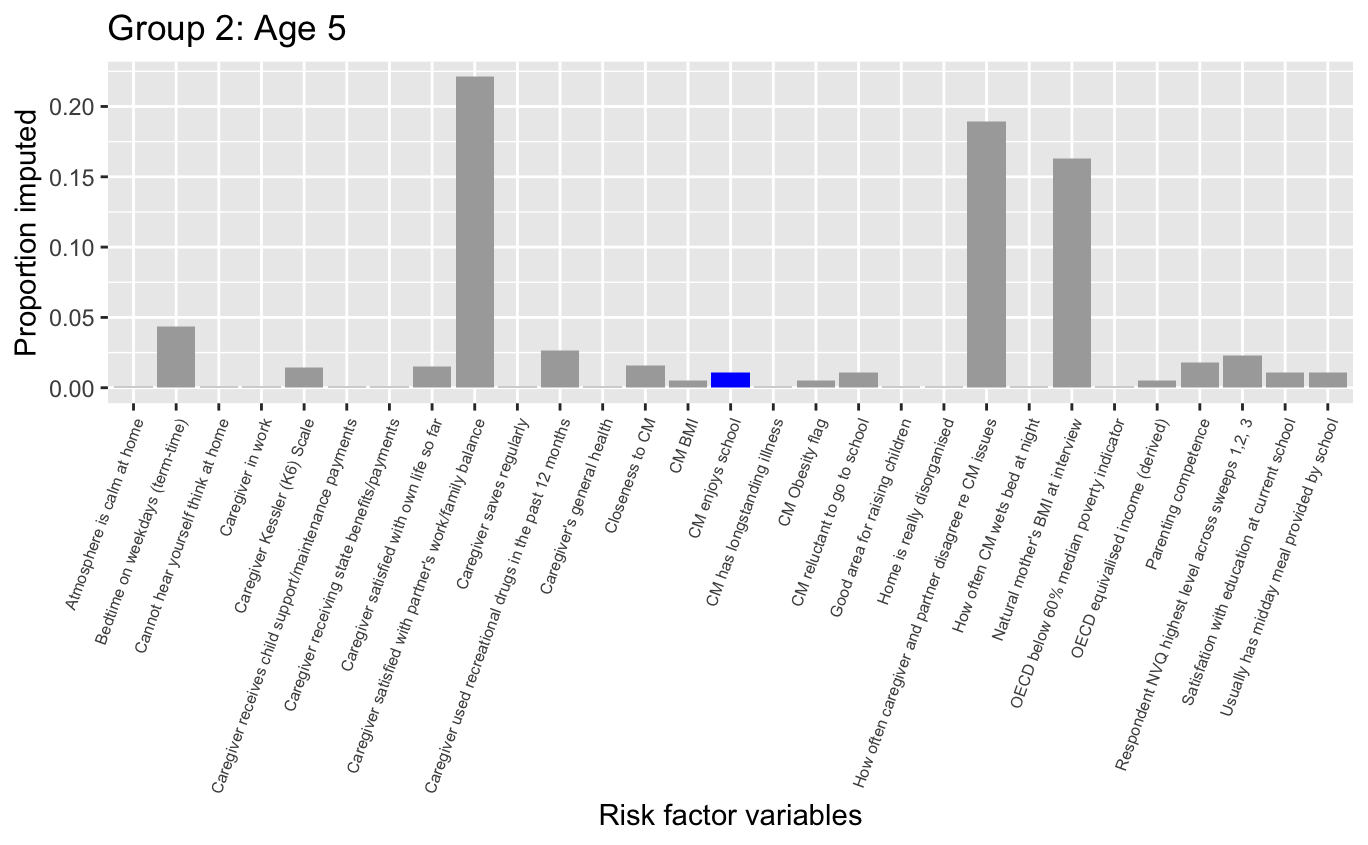


***Figure S2A*** *shows the proportion of risk factor variables imputed for Group 1 (with psychopathology) and* ***Figure S2B*** *for Group 2 (without psychopathology) from Age 5. Significant risk factors that were validated are highlighted in blue.*

**3A**


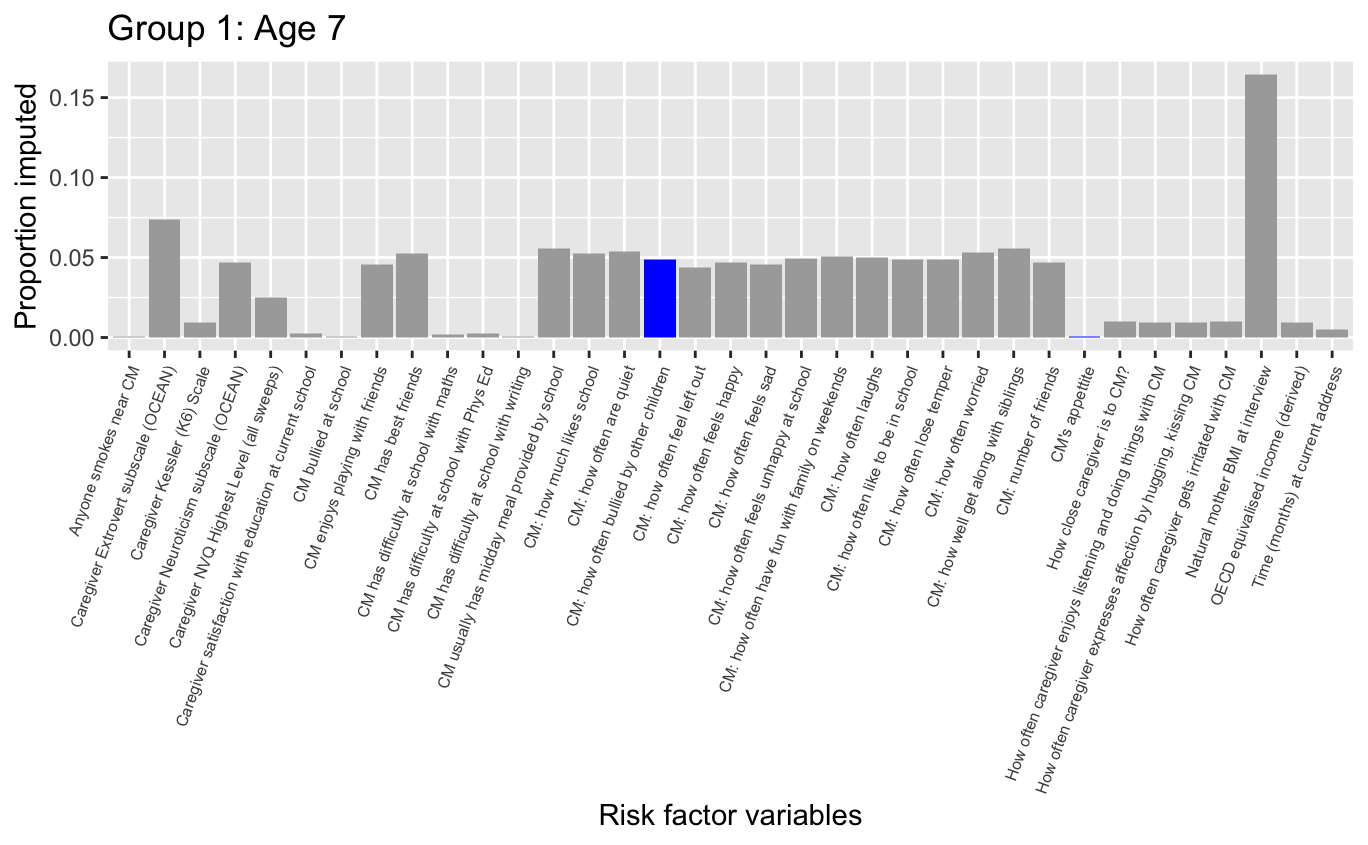


**3B**


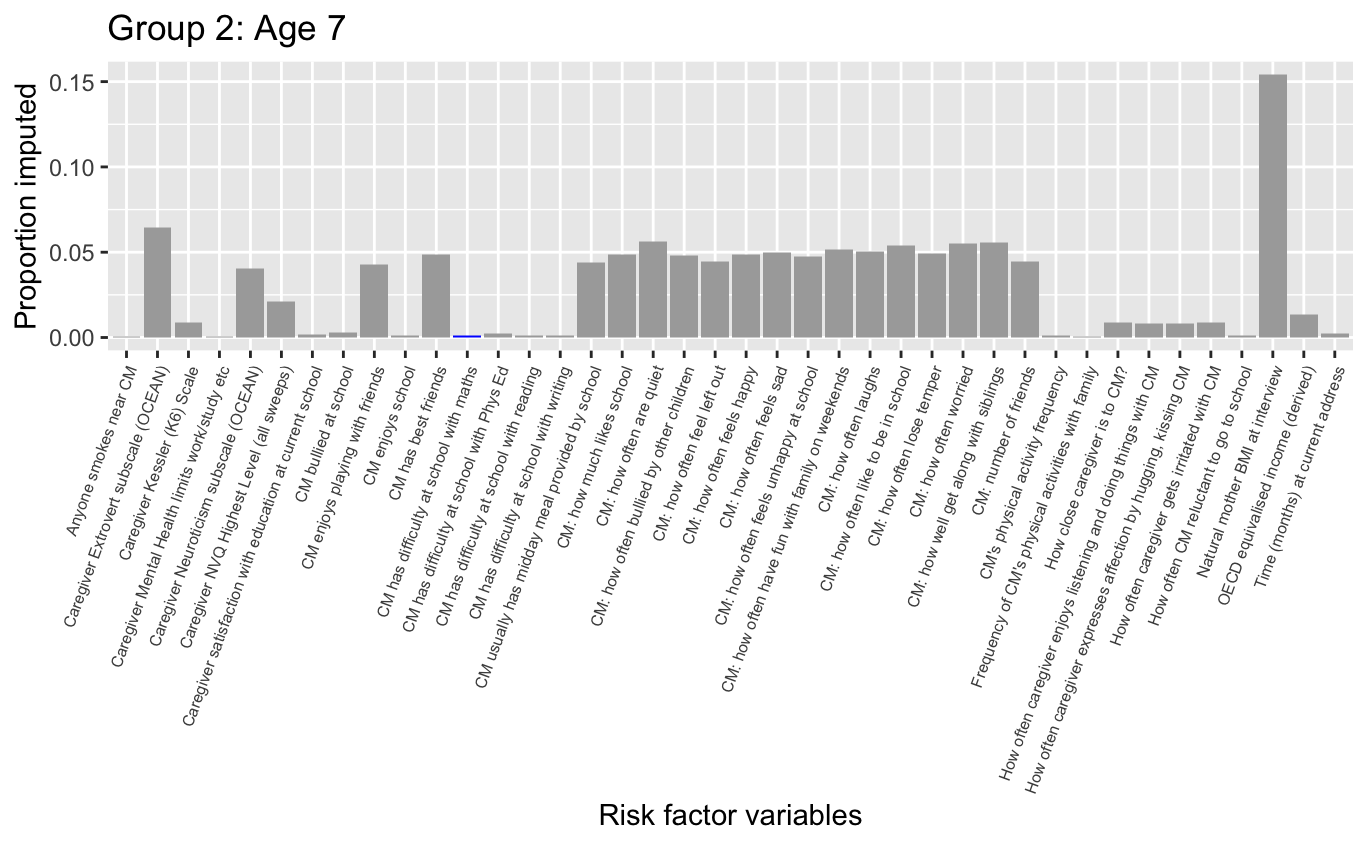


***Figure S3A*** *shows the proportion of risk factor variables imputed for Group 1 (with psychopathology) and* ***Figure S3B*** *for Group 2 (without psychopathology) from Age 7. Significant risk factors that were validated are highlighted in blue.*

**4A**


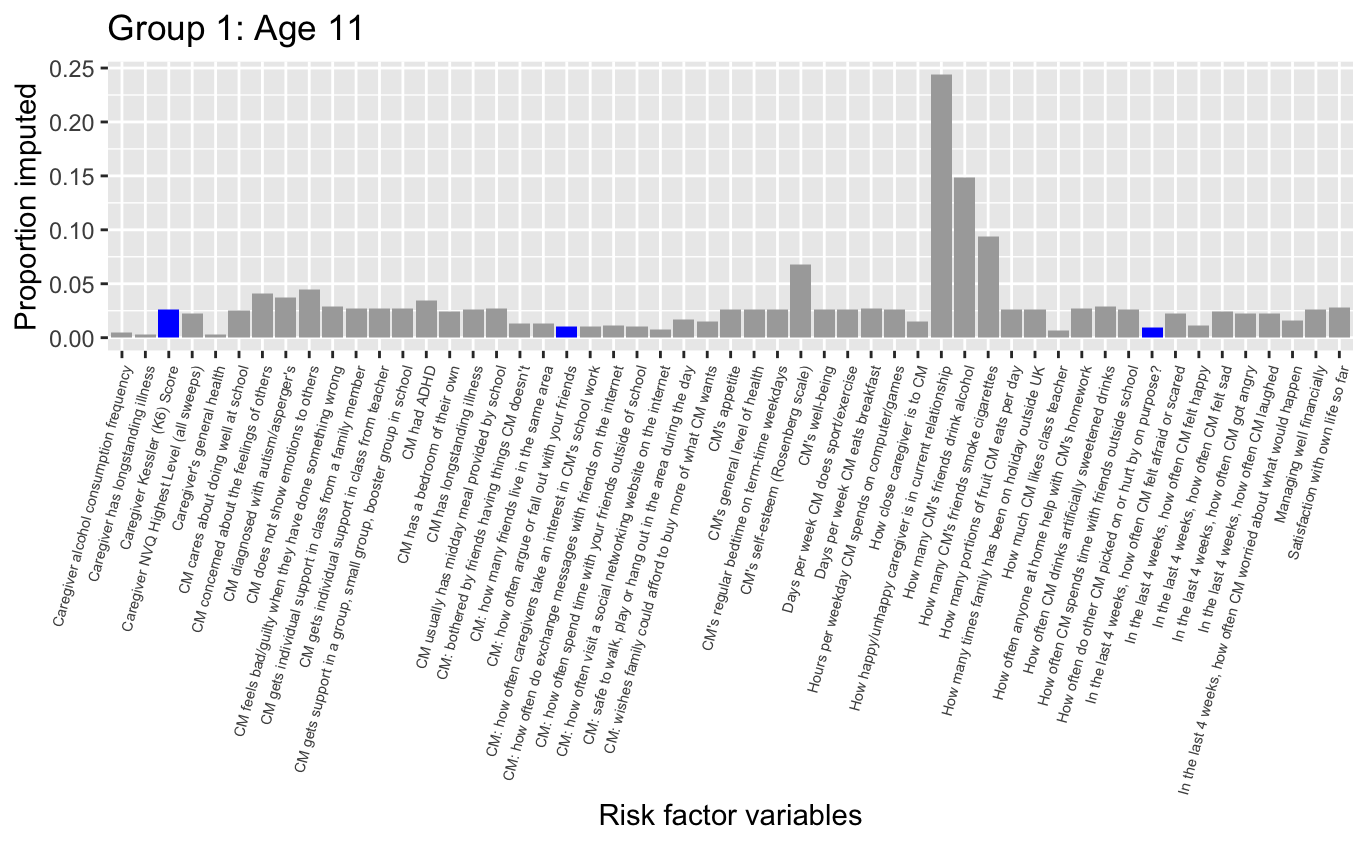


**4B**


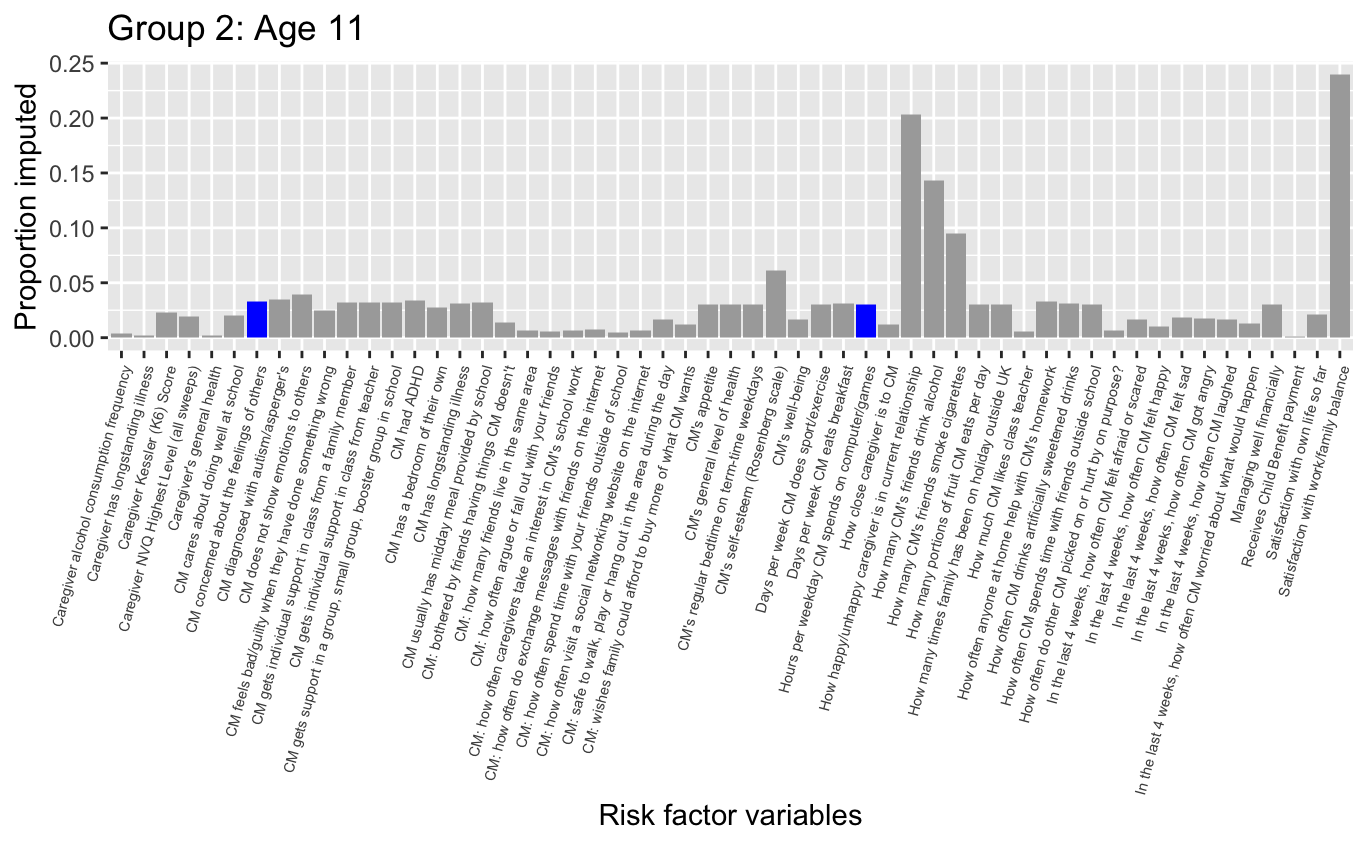


***Figure S4A*** *shows the proportion of risk factor variables imputed for Group 1 (with psychopathology) and* ***Figure S4B*** *for Group 2 (without psychopathology) from Age 11. Significant risk factors that were validated are highlighted in blue.*

**5A**


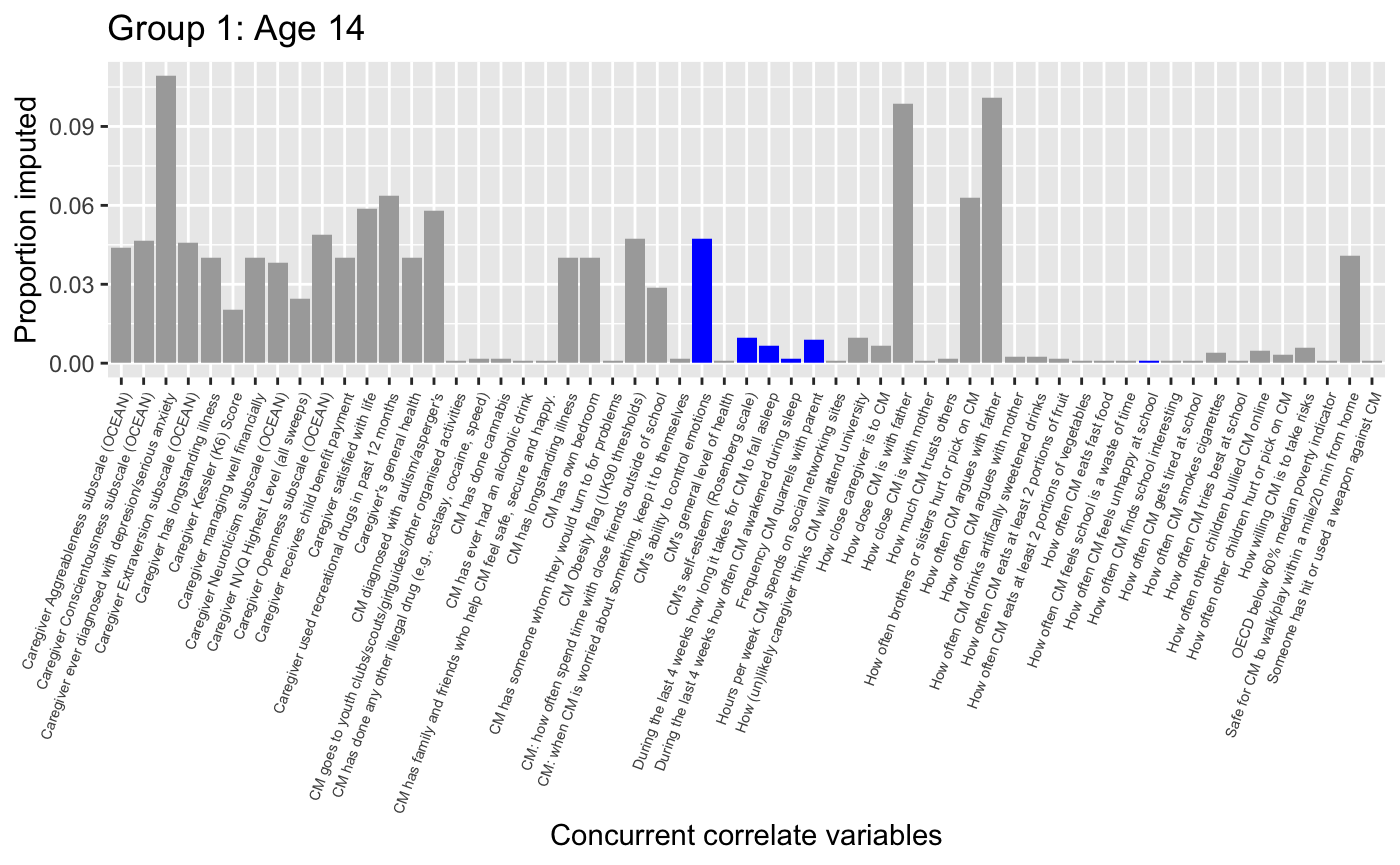


**5B**


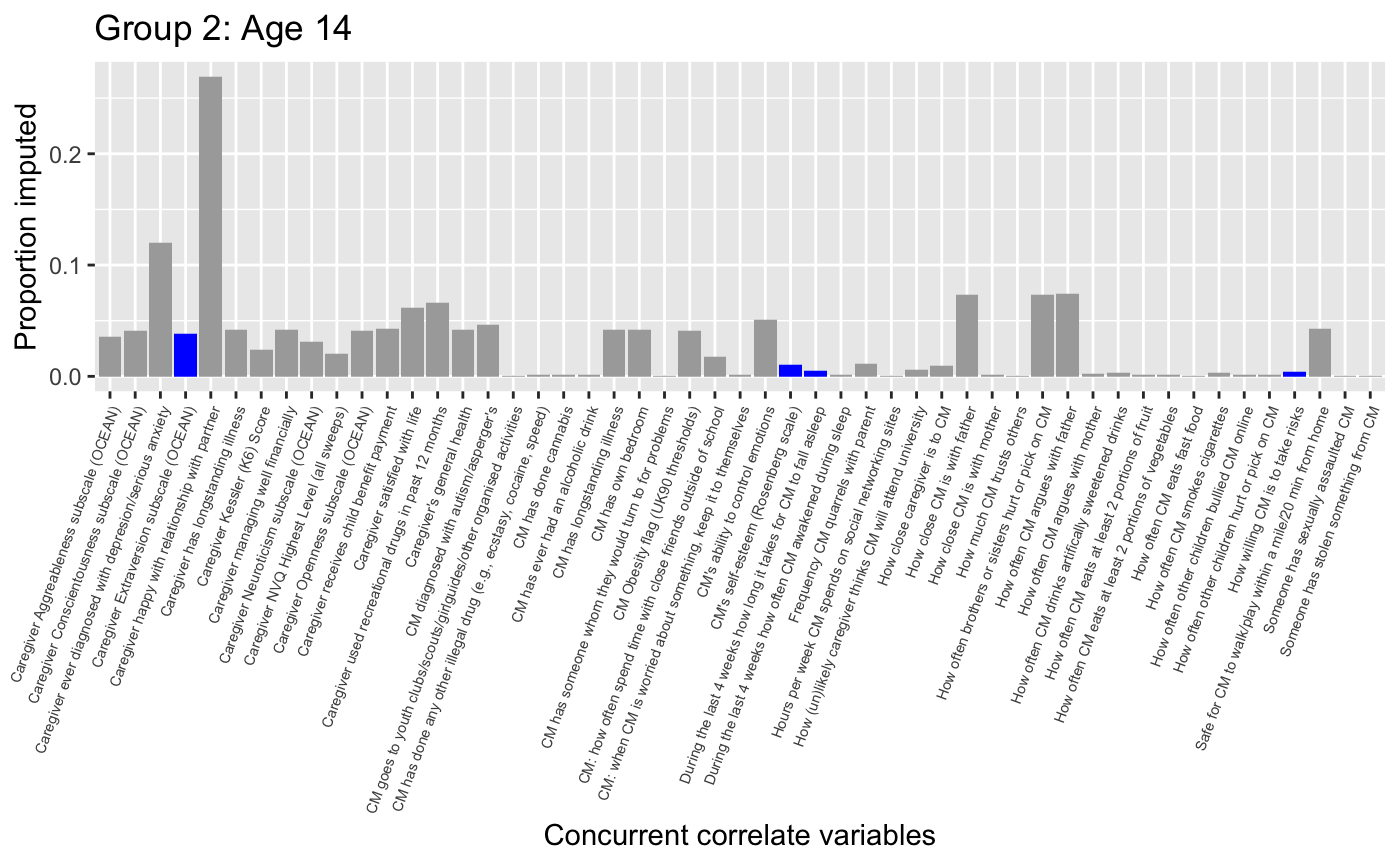


***Figure S5A*** *shows the proportion of concurrent correlate variables imputed for Group 1 (with psychopathology) and* ***Figure S5B*** *for Group 2 (without psychopathology) from Age 14. Significant concurrent correlates that were validated are highlighted in blue.*
